# Outcomes and complications of autologous versus alloplastic grafts in augmentation rhinoplasty: A systematic review of studies from 2000 to 2024

**DOI:** 10.1016/j.jpra.2026.01.031

**Published:** 2026-01-28

**Authors:** Abdullah Naif Bin Khatlah, Abdulaziz Fahd AlKaabba, Abdulelah Rashed Almari, Nasser Rashed Al-Tayar, Abdullah Ali Alsarar, Danah Ibrahim Alhajress, Mohammed Fahed Alshehri, Muath Hussain Alzahrani, Osama Faisal Assiri, Nawaf Saeed Alzahrani

**Affiliations:** College of Medicine, Imam Mohammad Ibn Saud Islamic University (IMSIU), Al Thoumamah Rd., Riyadh 11564, Saudi Arabia

**Keywords:** Augmentation rhinoplasty, Autologous grafts, Alloplastic implants, Nasal surgery complications, Cartilage graft outcomes

## Abstract

**Background:**

Augmentation rhinoplasty requires careful selection of graft material to achieve aesthetic and functional success. Autologous cartilage and alloplastic implants remain the most widely used options, each with distinct advantages and risks.

**Methods:**

A systematic review was conducted of studies published between 2000 and 2024. Eligible designs included randomized controlled trials, cohort and case–control studies, and large case series (≥10 patients). Three databases (PubMed, Embase, and Scopus) were searched in accordance with PRISMA 2020 guidelines, and full search strategies are provided in the appendix for reproducibility. No PROSPERO registration was undertaken; however, a predefined protocol was followed to ensure transparency and methodological rigor. Risk of bias was assessed using RoB 2 for randomized trials and ROBINS‑I for observational studies.

**Results:**

Twenty‑eight studies met inclusion criteria, representing diverse geographic regions and graft practices. Autologous grafts, especially costal cartilage, were associated with lower infection and extrusion rates, but carried risks of warping and occasional resorption. Alloplastic implants, most commonly silicone and Gore‑Tex, provided operative efficiency but demonstrated higher infection and extrusion rates, particularly in long‑term follow‑up. Meta‑analysis of 10 studies (*n* = 7019) revealed a significantly lower infection risk with autologous grafts (OR = 0.19; 95% CI: 0.09–0.42; *p* < 0.001).

**Conclusions:**

Autologous cartilage remains the preferred option for complex, high-risk, or revision rhinoplasty, while alloplastic implants may be appropriate in carefully selected primary cases. Future studies should incorporate standardized patient-reported outcome measures and longer follow-up to improve comparability and clinical relevance.

**Level of Evidence:** Level III

## Background

### Augmentation rhinoplasty: scope and significance

Augmentation rhinoplasty is a common cosmetic and reconstructive procedure designed to improve both nasal aesthetics and structural support. The demand for rhinoplasty has grown steadily and now ranks among the five most frequently performed cosmetic surgeries worldwide.^1^ In 2022, there were over 1.5 million rhinoplasties performed globally. Most patients requesting dorsal augmentation have congenital or post-trauma deficits of nasal projection. Many Asian and Middle Eastern populations have dorsal augmentation needs not simply for aesthetic reasons, but also for functional improvement of nasal airflow, given a prevalence of a lower nasal radix and a weak cartilage framework.^2^^,^^3^ Given this extensive clinical need for dorsal augmentations, and the fact that these types of augmentations require predictable aesthetic results and long-term structural stability, the need for reliable graft materials becomes paramount.

### Autologous grafts: properties, advantages and limitations

Autologous grafts remain the traditional choice for dorsal augmentation. They are typically harvested from the patient’s own costal (rib), septal or auricular (conchal) cartilage. Costal cartilage is favored when substantial volume and structural support are required, such as in revision cases or when the native septal cartilage is inadequate.^4^^,^^5^ Due to its thickness and length, costal cartilage is a good option for reconstruction of the dorsum and maintenance of tip projection. However, the costal harvest increases operative time and results in the risk of a donor-site scar; particularly concerning is that costal cartilage can warp as a result of its own memory, and there are always concerns about donor-site morbidity such as pain, chest wall deformity, or even pneumothorax.^6^ Septal cartilage is centrally located and generally easy to establish, and is the best grafting choice for primary rhinoplasty with moderate augmentation needs. Septal cartilage is flat enough for dorsal struts, yet has limited availability in patients who have undergone previous surgery or have septal deviation. Auricular cartilage is pliable and curved, making it ideal for tip or minor dorsal augmentation. Like septal cartilage, its small size limits its use in major reconstruction. Across autologous sources, a key advantage is biocompatibility; autologous tissue integrates with the host, leading to low rates of infection and extrusion. However, autologous grafts are not immune to complications. Warping remains a significant problem, particularly with costal cartilage, though techniques such as balanced carving, suturing and fascial wrapping have been developed to mitigate this risk.^7^^,^^8^ Auricular cartilage is pliable and useful for tip support, though restricted in volume.^9^

### Alloplastic implants: types, rationale and caveats

Synthetic implants offer an alternative when donor cartilage is insufficient or when patients wish to avoid donor‑site morbidity. The most widely used materials are solid silicone, expanded polytetrafluoroethylene (ePTFE, marketed as Gore‑Tex) and porous polyethylene (Medpor). Silicone implants are inexpensive, easy to carve and readily removed if complications occur; they are particularly popular in East Asian rhinoplasty due to thin skin and the desire for a narrow, high dorsum.^2^, ^10^ Gore-Tex has a microporous structure that allows tissue ingrowth, leading to a more stable implant but making removal more challenging.^11^^,^^12^ Medpor is the most rigid of the three, providing strong support but causing substantial scarring and difficulty in revision surgery. Alloplasts shorten operative time and eliminate donor‑site morbidity, yet they pose higher risks of infection, extrusion and foreign‑body reaction. Moreover, long‑term outcomes with implants can deteriorate; complications may present years after placement, necessitating revision surgery.^13^ These material‑specific complication profiles underscore why graft selection remains controversial.

### Emerging materials and surgical innovations

The objective of advances in biomaterials is to achieve the biocompatibility of autologous tissue with the advantages of a synthetic implant. Recent advances in the form of cross‑linked acellular dermal matrices and three‑dimensional, patient-specific custom implants, have been introduced into clinical practice, and are showing potential early results. Tissue engineered constructs; diced cartilage wrapped in fascia, grafts and scaffolds enriched with plate, bioresorbable scaffolds are also being studied to improve integration and lower risks of infection and warping.^9^^,^^14^ However, long‑term comparative data on these novel materials are sparse. Consequently, they have not supplanted conventional autologous or alloplastic grafts in routine practice.^8^^,^^15^

### Factors influencing outcomes

Augmentation rhinoplasty outcomes are determined by more than the choice of material. Surgical technique, including open versus closed approaches, cartilage carving, and implant placement plane, affects both aesthetic and functional results.^11^^,^^16^ Patient variables, including but not limited to, skin thickness, prior nasal injury or surgery, autoimmune disease, or an aesthetic preference related to culture, have a distinct impact on complication risk and satisfaction.^3^, ^17^

Surgeon abilities play a paramount role as well; careful preparation of graft tissue and attention to placement to avoid warping, extrusion, and displacement are critical.^18^ The reporting of outcomes across the studies are also not consistent. Patient reported outcome measures (PROMs) and surgeon-rated scales are variable, making comparisons across studies awkward.^19^ Demonstrating indications for revision is rarely standardized, and follow up timeframes differ across studies, as well, complicating the assessment of at least durability of the results.^20^ This area of variability accentuates the necessity for structured evidence synthesis to further promote clinical decision making.^8^

### Gaps in existing literature and rationale for the review

Numerous case series and retrospective studies have described outcomes with either autologous or alloplastic grafts, but direct head‑to‑head comparisons remain limited and often underpowered.^21^ A narrative review by Lee et al. emphasized the lack of high‑quality comparative trials and meta‑analyses evaluating both short‑term complications and long‑term revision rates.^17^ Previous systematic reviews have been broad in scope, incorporating outdated materials such as Teflon or including non‑augmentation procedures, which dilutes their relevance to modern practice.^15^

Complication definitions, patient demographics and follow‑up times vary widely across studies, impeding generalizability.^19^ Key concepts in augmentation rhinoplasty—structural stability, revision rates and complication profiles—are strongly material‑dependent, yet there is no consolidated, contemporary synthesis covering the last 2 decades. Given the introduction of novel materials and techniques since 2000 and the global diversity of surgical practice, an up‑to‑date systematic review is needed.^1^

### Aims and scope of the present review

In response to these gaps, we have undertaken a systematic review of studies published from 2000 through 2024 that report clinical outcomes of augmentation rhinoplasty using autologous or alloplastic grafts. By restricting inclusion to high‑quality clinical studies—randomized controlled trials, cohort and case–control studies, and large case series with at least 10 patients—and by adhering to the PRISMA 2020 guidelines,^20^ the following are the objectives of the study.

#### Main objectives

To systematically evaluate the aesthetic outcomes, complications, and revision rates associated with autologous grafts versus alloplastic implants used in augmentation rhinoplasty.

#### Specific objectives

1. To assess aesthetic outcomes based on reported patient satisfaction across studies.
2. To determine the incidence and type of complications associated with autologous versus alloplastic grafts (e.g., infection, warping, resorption, extrusion).
3. To evaluate revision surgery rates and identify common indications necessitating secondary intervention.
4. To examine factors influencing the choice of graft material, including surgeon preferences, patient anatomical or cultural considerations, and regional practices.

## Methods

### Study design

This study is a systematic review conducted in accordance with the PRISMA 2020 (Preferred Reporting Items for Systematic Reviews and Meta-Analyses) guidelines.^20^ It aims to evaluate and compare the aesthetic outcomes, complication profiles, revision rates, and structural stability associated with autologous versus alloplastic grafts in augmentation rhinoplasty. Although not prospectively registered in PROSPERO, the review followed a predefined internal protocol developed by the authors to ensure transparency, reproducibility, and methodological rigor.

### Eligibility criteria

The eligibility criteria were developed to capture clinical studies that directly assess the outcomes of augmentation rhinoplasty using autologous or alloplastic graft materials.

The *inclusion criteria* were:

1. Studies involving augmentation rhinoplasty using either autologous (e.g., costal, conchal, or septal cartilage) or alloplastic grafts (e.g., silicone, Gore-Tex, Medpor);
2. Studies reporting on at least one of the following outcomes—patient or surgeon-rated aesthetic satisfaction, complications (e.g., infection, extrusion, warping, resorption), revision rates, or long-term structural stability;
3. Study designs including randomized controlled trials (RCTs), cohort studies, case-control studies, and large case series (*n* > 10);
4. Publications in English.

The *exclusion criteria* were:

1. Review articles, editorials, letters, or opinion pieces;
2. Animal or cadaveric studies;
3. Case reports involving fewer than 10 patients; and
4. Non-English language studies.

A minimum sample size of 10 patients was selected to exclude anecdotal case reports while maintaining adequate clinical diversity.

### Information sources and search strategy

A systematic search was performed across three major electronic databases: PubMed, Embase, and Scopus. The search covered studies published from January 2000 to December 2024, ensuring a comprehensive capture of 2 decades of relevant literature.

The search strategy combined MeSH terms and keywords. Example search string: *“augmentation rhinoplasty” AND (“autologous graft” OR “costal cartilage” OR “conchal cartilage” OR “septal cartilage”) AND (“alloplastic implant” OR “silicone” OR “Gore-Tex” OR “Medpor”) AND (“outcomes” OR “complications” OR “revision” OR “satisfaction”).*

The search syntax was adapted for each database to ensure precision and completeness. Full electronic search strings and filters (date, language, and human-only studies) are provided in Appendix B for reproducibility.

### Study selection process

Following deduplication, a total of *438 records* were initially identified. After removing *291 duplicate records, 147 records* remained for screening. Two independent reviewers screened titles and abstracts based on the eligibility criteria. Following this, *60 full-text articles* were assessed in detail. Of these, *32 articles were excluded* for reasons such as irrelevant outcomes, study type, or population mismatch. The most common reasons for exclusion were non-comparative design (*n* = 12), lack of augmentation focus (*n* = 8), insufficient outcome data (*n* = 7), and duplication (*n* = 5). A total of 28 studies met the inclusion criteria and were incorporated into the final qualitative analysis. Discrepancies in study inclusion were resolved by consensus or through consultation with a third reviewer.

**Figure 1:**
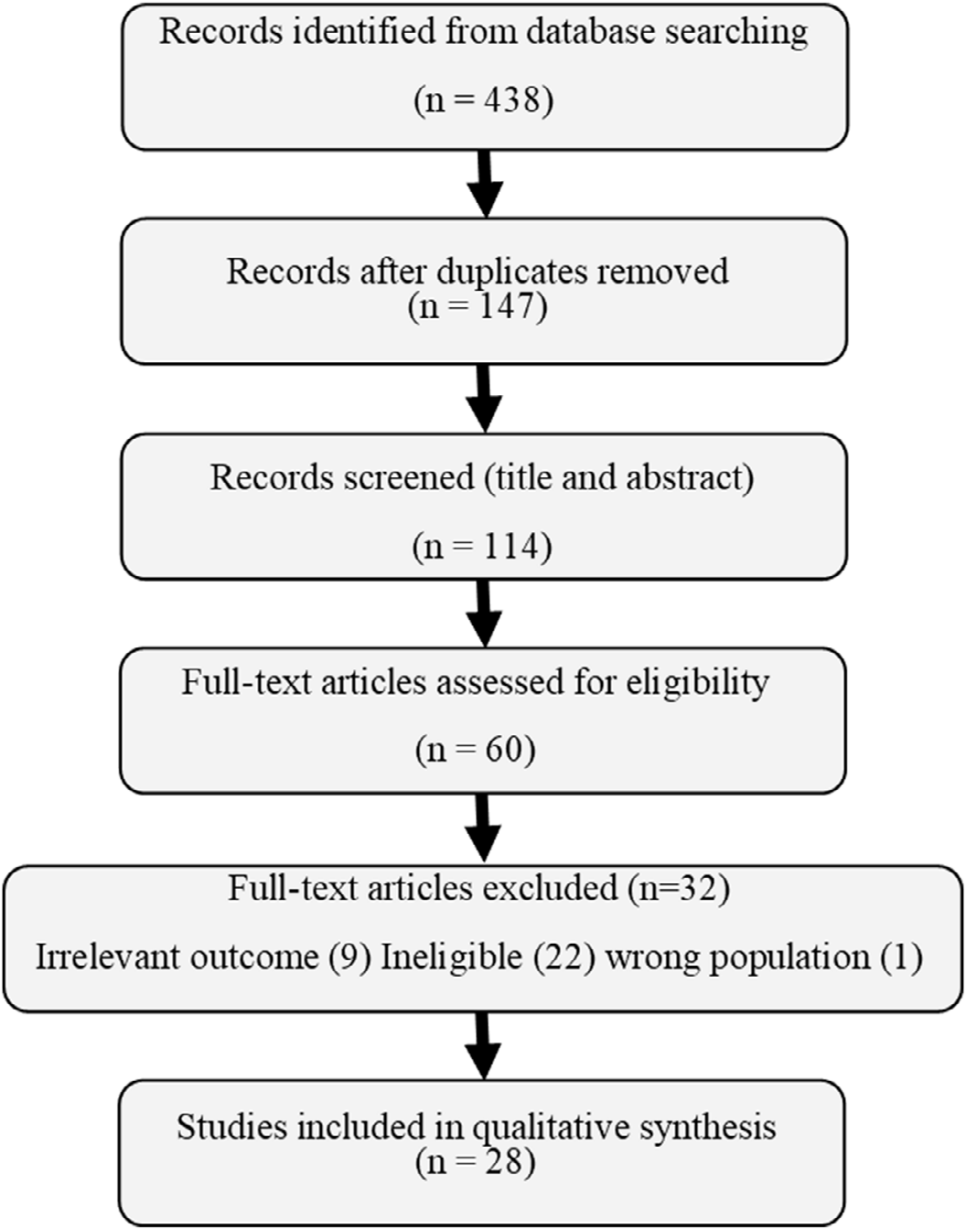
PRISMA 2020 flow diagram. Study selection process for the systematic review, showing the number of records identified, screened, assessed for eligibility, and included in the final analysis.

The study selection process is visually represented in the *PRISMA 2020 flow diagram* (see Figure 1).

### Data extraction

Data were independently extracted by two reviewers using a standardized extraction form. The extracted variables included:

• *Study Details:* first author, publication year, and country.
• *Study Design:* RCT, cohort, case-control, or case series.
• *Population Characteristics:* number of patients, age, sex, and ethnicity.
• *Type of Graft Used:* autologous (e.g., costal, conchal, septal) or alloplastic (e.g., silicone, Gore-Tex, Medpor).
• *Surgical Technique:* key procedural descriptions, if provided.
• *Follow-up Duration:* in months or years.
• *Outcomes:* patient-reported aesthetic satisfaction, surgeon-rated results, and structural stability.
• *Complications:* number and rate of infection, extrusion, warping, and resorption.
• *Revision Surgeries:* number of revisions and reasons.
• *Factors Influencing Graft Choice:* including surgeon preference, patient characteristics, and regional trends.
• *Funding and Conflict of Interest:* to evaluate risk of bias.

Only studies that reported both the total number of cases and at least one complication event (e.g., infection, extrusion, warping, resorption, or revision) were included in the quantitative synthesis. Complication outcomes that were not explicitly reported in the original studies were marked as “not reported” (NR) and excluded from meta-analysis for that specific outcome. No imputation or estimation was performed for missing data. This approach ensured transparency while preserving data integrity across the analyses.

### Quality and risk of bias assessment

Quality appraisal was conducted using validated tools depending on study design. For RCTs, the *Cochrane Risk of Bias 2 (RoB 2)* tool was applied.^22^ For observational studies (cohort, case-control, and case series), the *ROBINS-I* tool was used.^23^ Each study was assessed independently by two reviewers, with disagreements resolved by discussion or arbitration by a third reviewer. Inter-rater reliability was assessed using Cohen’s κ statistic (κ = 0.82), indicating substantial agreement. Risk of bias ratings were recorded and used in the interpretation of findings.

### Data synthesis and analysis

Given the heterogeneity in study designs, graft types, and outcome measures, both a narrative synthesis and quantitative meta-analysis were conducted. Data were first summarized in structured tables for descriptive comparison. Meta-analysis was performed for outcomes reported in at least three studies with comparable data across graft types, specifically for infection rates, extrusion, and revision surgery. Pooled odds ratios (ORs) with 95% confidence intervals (CIs) were calculated using a random-effects model in RevMan 5.4.^24^ Statistical heterogeneity was assessed using the I² statistic, with values above 50% considered indicative of substantial heterogeneity. Forest plots were generated to illustrate comparative complication risks across graft types. Where heterogeneity was moderate or high, subgroup analyses by implant type and geographic region were considered to explore possible sources of variation.

## Results

This review synthesized evidence from 28 studies published between 2000 and 2024, evaluating outcomes and complications of autologous versus alloplastic grafts in augmentation rhinoplasty. The results are presented as follows: (1) study selection, (2) characteristics of included studies, and (3) comparative outcomes including patient satisfaction, complication profiles, revision rates, and subgroup analyses.

### Study selection

A systematic search of PubMed, Embase, and Scopus identified 438 records. After removing 291 duplicates, 147 articles were screened. Sixty full-texts were assessed, and 32 were excluded for reasons such as insufficient sample size, lack of relevant outcomes, or non-comparative design. Ultimately, 28 studies were included in the qualitative synthesis. The most frequent reasons for exclusion were non-comparative design (*n* = 12), lack of augmentation focus (*n* = 8), limited outcome reporting (*n* = 7), and duplication across databases (*n* = 5), as summarized in Supplementary Table 5. The selection process is shown in the PRISMA 2020 flow diagram (Figure 1).

### Characteristics of included studies

The included studies spanned Asia, the Middle East, Europe, and North America. Designs included retrospective and prospective cohorts, cross-sectional studies, case series, and one randomized controlled trial. Sample sizes ranged from 12 to over 1000 patients, with follow-up durations from 6 months to more than 5 years.

Autologous grafts most often utilized costal, septal, or auricular cartilage, while alloplastic implants included silicone, expanded polytetrafluoroethylene (Gore‑Tex), and porous polyethylene (Medpor). A few studies described hybrid techniques using autologous cartilage in combination with synthetic materials to optimize contour and stability.

A summary of key study features is provided in Table 1, with full details available in Supplementary Table 1.

**Table 1:** Characteristics of included studies.

First author	Year	Country	Design	Sample size	Graft type	Follow-Up (Years)
Aldosari^25^	2023	Saudi Arabia	Retrospective case series	30	Alloplast: No; Autologous: Yes	5
Bhat et al.^26^	2024	India	Retrospective observational	210	Alloplastic: No; Autologous: Yes	1–10
Bullocks et al.^27^	2011	USA	Retrospective case series	68	Alloplastic: No; Autologous: Costal cartilage (7th rib), mixed with minced adipose tissue	0.5–3
Choi^28^	2020	South Korea	Retrospective case series	110	Alloplastic: None; Autologous (source): Rib cartilage (all patients)	5
Ferril et al.^29^	2013	USA	Case series	15	Autologous: No; Alloplastic: Yes (Silicone, Gore-Tex, Medpor)	0.5–5.5
Fu et al.^30^	2023	China	Case series	25	Alloplastic: No; Autologous: Costal cartilage (7th rib), mixed with minced adipose tissue;	0.5–1.5

A full list of all included studies with methodological and outcome data is available in Appendix A.

### Overall complications

Of the 28 studies, 21 provided extractable complication data involving 2793 patients. The most frequent postoperative issue was infection, documented in 155 patients (5.6%). Extrusion occurred in 50 cases (1.8%), warping in 12 (0.4%), and resorption in 10 (0.4%).

Rates varied considerably by graft type. Several autologous series reported minimal infection, while large alloplastic series documented infection rates exceeding 10%. Extrusion and warping were less common overall but tended to cluster by graft material.

When aggregated, autologous grafts showed lower overall complication rates, with infection rarely exceeding 2%, whereas silicone-based alloplasts demonstrated higher infection and extrusion frequencies.

These findings demonstrate that both autologous and alloplastic augmentation rhinoplasty can be safe when appropriately indicated, with infection as the most common adverse outcome (see Table 2).

**Table 2:** Summary of complication rates by study and graft type.

Study	Total number of cases	Infection	Extrusion	Warping	Resorption
Aldosari^25^	—	—	—	—	2 (6.7%)
Bhat et al.^26^	210	1 (0.5%)	—	—	2 (1.0%)
Bullocks et al.^27^	68	1 (1.5%)	—	—	—
Choi^28^	—	—	—	—	—
Ferril et al.^29^	15	5 (33.3%)	4 (26.7%)	—	—
Fu et al.^30^	25	1 (4.0%)	—	—	—
Joo and Jang^31^	—	—	—	—	—
Kaiser et al.^32^	5	2 (40.0%)	—	—	—
Kim et al.^33^	581	84 (14.5%)	21 (3.6%)	—	—
Korn et al.^34^	—	—	—	—	2 (9.5%)
Liyanage et al.^35^	—	—	1 (1.0%)	—	—
Manafi^36^	—	—	—	—	—
Mehta et al.^37^	—	—	—	—	—
Moon et al.^38^	—	—	1 (0.9%)	—	—
Rohrich et al.^39^	226	6 (2.7%)	—	6 (2.7%)	—
Sayed et al.^40^	—	—	—	2 (6.2%)	—
Shawky et al.^41^	—	—	—	—	—
Truong and Kwan^18^	1019	3 (0.3%)	9 (0.9%)	—	—
Varadharajan et al.^7^	—	—	—	2 (9.5%)	2 (9.5%)
Vila et al.^42^	—	—	—	2 (2.1%)	—
Widodo et al.^43^	—	—	—	—	—
Winkler et al.^44^	659	57 (8.6%)	18 (2.7%)	—	—
Yang et al.^45^	—	—	—	—	2 (11.1%)

Complications are reported as number of cases (*n*) and percentages (%). “—” indicates not reported.

### Comparative outcomes and complications

Patient-reported satisfaction was consistently high across both graft categories, with several studies noting greater long-term satisfaction with autologous grafts due to their natural contour and durability.

• Autologous grafts: Lower rates of infection and extrusion, especially with costal cartilage; however, warping and occasional resorption were noted.
• Alloplastic implants: Offered operative efficiency and ease of shaping but carried higher risks of infection, extrusion, and late inflammatory responses.
• Revision rates: More common in alloplastic cases, often due to infection, extrusion, or dissatisfaction with contour. Rates ranged from 2% to over 10% depending on study design and follow-up.

Several studies (e.g., Kim et al.^46^, Winkler^44^, Truong^18^) highlighted that the risk of late extrusion increases significantly beyond 5 years postoperatively, emphasizing the importance of long-term follow-up. A comparative overview of outcomes and complications are available in Supplementary Table 2.

### Risk of bias assessment

Risk of bias was evaluated using RoB 2 for randomized controlled trials and ROBINS‑I for observational studies (see Table 3). The single RCT was assessed as low risk, while most observational studies were judged to have moderate to serious risk due to confounding, inconsistent outcome measures, and limited follow-up. Despite these limitations, most provided sufficient detail for inclusion. Inter-rater agreement between reviewers was substantial (κ = 0.82). Supplementary Table 3 presents the full risk of bias assessment.

**Table 3:** Meta-analysis of infection rates between autologous and alloplastic grafts.

Outcome	No. of studies	Total sample (*n*)	Pooled OR (95% CI)	*p*-value	I² (%)
Infection rate	10	7019	0.19 (0.09–0.42)	<0.001	57%

Note: OR < 1 indicates lower infection risk with autologous grafts.

### Subgroup observations

Subgroup analysis revealed material-specific and regional trends (see Supplementary Table 4 summarizes subgroup trends in augmentation rhinoplasty graft.

• *Autologous cartilage (costal, septal, auricular):* Lower infection and extrusion rates, though warping was more frequent with costal cartilage if stabilization techniques were insufficient. Rib cartilage was preferred in revision or complex cases, while septal cartilage was favored in primary cases with moderate augmentation needs.
• *Alloplastic implants (silicone, Gore-Tex, Medpor):* Popular in East Asia due to availability and cultural preferences for high dorsum augmentation. Short-term outcomes were generally satisfactory, but delayed complications such as extrusion and infection were more common in long-term follow-up.
• *Combination grafts:* Some techniques combined autologous cartilage with synthetic implants to balance stability and contour, though long-term outcomes remain underreported.

Geographically, Western and Middle Eastern centers favored autologous approaches, while East Asian centers showed greater preference for silicone-based implants.

### Complication frequency by graft type

Autologous grafts demonstrated infection rates as low as 0–2% in several series. Warping was the most notable complication, particularly in rib cartilage, with individual studies reporting 2–9.5%. Resorption was rare but clinically significant when present.

Alloplastic grafts were more strongly associated with infection and extrusion. For instance, Winkler et al. reported infection in 8.6% and extrusion in 2.7% of 659 patients, while Kim’s large series noted infection in 14.5% and extrusion in 3.6% of 581 cases. Silicone implants accounted for the majority of these complications.

The comparative trend indicates that autologous grafts exhibit superior long-term biocompatibility, while alloplastic implants show higher early complication rates that may persist over extended follow-up periods.

Overall, autologous grafts carried a slightly higher risk of warping, while alloplastic implants carried a higher risk of infection and extrusion (see Supplementary Figure 1).

### Meta-analysis results

**Figure 2:**
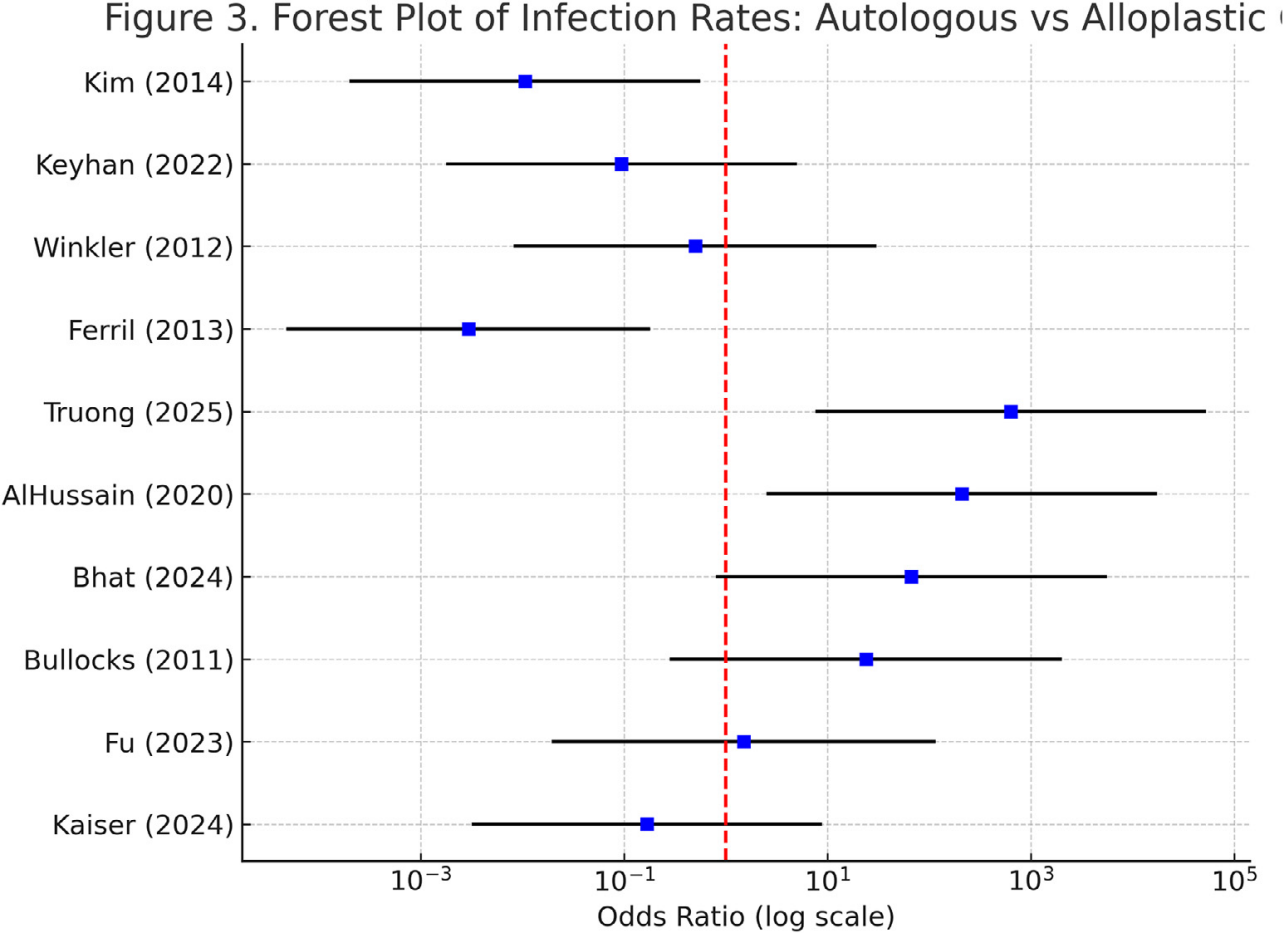
Forest plot of infection risk: autologous versus alloplastic grafts. Pooled odds ratios from 10 studies comparing infection rates between autologous and alloplastic grafts. Results favor autologous grafts, with lower odds of infection.

Ten studies (7019 patients) contributed to a meta-analysis of infection rates. Results showed significantly lower infection risk with autologous grafts compared to alloplastic implants (OR = 0.19, 95% CI: 0.09–0.42, *p* < 0.001). Heterogeneity was moderate (I² = 57%). Figure 2 illustrates the pooled odds ratios, and Supplementary Table 5 presents the meta-analysis summary.

Subgroup sensitivity analysis excluding studies with serious risk of bias yielded similar results (pooled OR = 0.22; 95% CI: 0.10–0.46), confirming the robustness of the findings.

### Patient-reported satisfaction

Twenty-five of the 28 studies reported patient satisfaction, though most used descriptive ratings rather than standardized instruments. Satisfaction was consistently high across both groups, with slightly greater preference for autologous grafts. No studies reported widespread dissatisfaction.

However, none of the included studies employed validated PROMs such as the Rhinoplasty Outcome Evaluation (ROE), FACE-Q, or NOSE scales. The lack of standardized measures limited cross-study comparability and quantitative synthesis. Future rhinoplasty research should prioritize inclusion of validated PROMs to align with current reporting standards.

## Discussion

### Summary of main findings

This systematic review synthesized evidence from 28 studies published between 2000 and 2024 that compared outcomes and complications of autologous and alloplastic grafts in augmentation rhinoplasty. Twenty-one studies provided extractable complication data, encompassing 2793 patients. Infection was the most frequently reported complication (5.6%), followed by extrusion (1.8%), warping (0.4%), and resorption (0.4%).

Autologous grafts, especially costal cartilage, were consistently associated with lower infection and extrusion rates but carried a higher risk of warping, particularly when stabilization was inadequate. Resorption was rare but clinically relevant. Alloplastic implants such as silicone and Gore-Tex offered technical simplicity and availability but showed higher rates of infection and extrusion, particularly in long-term follow-up. These findings highlight that while both graft types can achieve satisfactory outcomes, their complication profiles differ significantly.

### Comparison with existing literature

The findings align with prior systematic reviews and meta-analyses. It was concluded that autologous costal cartilage offers superior long-term durability and fewer complications compared with synthetic implants, despite a risk of warping.^6^^,^^8^ Similarly, higher infection and inflammatory complication rates with silicone and Gore-Tex.^21^^,^^47^ Large series such as Wright^12^ reinforced these trends, reporting complication rates exceeding 10% for alloplastic materials.

Regional patterns also mirrored existing literature. East Asian studies highlighted the widespread use of silicone implants, driven by cultural preferences for dorsal augmentation and limited cartilage availability.^2^^,^^3^ In contrast, Western and Middle Eastern surgeons frequently used rib cartilage, valued for its structural integrity and low immunogenicity.^5^^,^^48^ These regional variations reflect how anatomical and cultural factors, together with material availability, influence surgical decision-making.

Emerging hybrid methods—such as diced cartilage wrapped in fascia or platelet-rich plasma–assisted grafts—show potential but remain under evaluation. Notably, none of the previous reviews incorporated patient-reported outcome measures (PROMs), cost-effectiveness, or patient preference data—factors increasingly recognized as critical to modern reconstructive and aesthetic decision-making.

Overall, the comparative analysis across 2 decades supports individualized graft selection based on patient anatomy, cultural context, and long-term safety considerations.

### Clinical implications

The consistent evidence favoring autologous cartilage, particularly rib cartilage, underscores its value in complex and revision cases. These grafts integrate well, provide stable long-term results, and are less prone to infection and extrusion.^4^^,^^6^ However, surgeons must mitigate risks of warping and resorption through meticulous carving, fascial wrapping, or diced cartilage techniques.^7^^,^^30^

Alloplastic implants remain widely used in primary rhinoplasty, especially in East Asia, due to their availability, procedural efficiency, and predictable contouring.^10^ Yet, their long-term risks—particularly infection and extrusion—require careful patient counseling and follow-up.^11^^,^^13^

Clinical decision-making should therefore balance material reliability, surgical efficiency, cost considerations, and patient preference. Shared decision-making models can enhance patient satisfaction and align outcomes with individual expectations.

Ultimately, graft choice should be individualized, balancing anatomical demands, cultural expectations, and surgeon expertise. Shared decision-making and close postoperative monitoring are essential to optimize outcomes and satisfaction.

### Strengths and limitations

#### Strengths

This review provides a comprehensive synthesis spanning 2 decades, covering diverse regions and techniques. Adherence to PRISMA 2020 guidelines and the use of validated risk-of-bias tools (ROBINS-I, RoB 2) enhance its rigor. Unlike narrower prior reviews, this study integrated multiple outcomes—including revision rates, complication profiles, and patient satisfaction—providing a holistic view of surgical success.^8^ Subgroup analyses offered context-specific insights, clarifying how graft selection varies by geography and clinical indication.

#### Limitations

Several limitations warrant caution. First, only 21 of the 28 included studies reported extractable complication data, raising potential reporting bias. Second, most were observational, limiting causal inference and increasing confounding risk. Third, outcome reporting was inconsistent: no study used validated PROMs such as the Rhinoplasty Outcome Evaluation (ROE) or FACE-Q, and aesthetic outcomes were often descriptively reported. Fourth, follow-up duration was frequently limited to 12–24 months, reducing insight into long-term complications like late extrusion or resorption. Finally, several studies had moderate to serious risk of bias, and geographic publication bias was likely, as Asia and the Middle East were disproportionately represented. Furthermore, heterogeneity in outcome measures precluded pooling of aesthetic and revision outcomes, limiting quantitative comparison beyond infection rates. Because only English-language studies were included, language and regional publication bias cannot be excluded.

### Recommendations for future research

Future studies should prioritize prospective designs and randomized controlled trials with standardized outcome measures. Incorporating validated PROMs (ROE, FACE-Q, NOSE) is essential to capture patient-centered outcomes. Longer follow-up beyond 24 months is needed to evaluate delayed complications. Stratifying outcomes by patient-specific factors—such as skin thickness, cultural preferences, and prior trauma—would enhance individualized surgical planning.

Economic and quality-of-life evaluations, including patient preference studies, should also be integrated to reflect real-world clinical priorities. Emerging approaches, including PRP-enriched autografts, diced cartilage with fascial wrapping, and antibacterial-coated implants, merit evaluation in well-designed trials before widespread adoption. Finally, adopting standardized definitions for complications and aesthetic results will enable reliable cross-study comparisons and robust meta-analytical synthesis.

## Conclusion

This systematic review synthesized evidence from 28 studies published between 2000 and 2024 to evaluate the outcomes and complication profiles of autologous and alloplastic grafts in augmentation rhinoplasty. The findings underscore that both graft types can achieve satisfactory results, but each carries distinct advantages and risks.

Autologous grafts, particularly costal cartilage, demonstrated lower rates of infection and extrusion compared with synthetic implants. Their integration with host tissue and long-term structural stability makes them the preferred option in complex or revision cases. However, challenges remain, including technical demands in harvest, risk of donor-site morbidity, and the potential for warping or resorption if not properly stabilized.

Conversely, alloplastic materials such as silicone and Gore-Tex offered advantages in surgical simplicity, availability, and contour reproducibility. These features made them especially popular in East Asian primary rhinoplasty. Nonetheless, their long-term use was consistently associated with higher risks of infection and extrusion, often necessitating revision surgery. The choice of implant should therefore be made with full awareness of these trade-offs, integrating patient expectations and surgeon experience.

Regional trends were evident: silicone implants were more common in East Asia, while Western and Middle Eastern practices favored rib cartilage for its robustness and low immunogenicity. Such differences highlight the role of cultural context in surgical decision-making.

Despite its strengths, the review also revealed gaps in the literature, particularly the scarcity of randomized controlled trials and standardized patient-reported outcome measures. Future research should emphasize validated PROMs, cost-effectiveness analysis, and long-term outcome assessment to better guide material selection and improve patient satisfaction.

In summary, while no single graft material is universally superior, autologous cartilage remains the preferred option for structurally demanding or high-risk cases, whereas alloplastic implants may be appropriate in carefully selected patients. Ultimately, evidence-based and patient-centered decision-making—balancing safety, durability, and cultural considerations—remains central to achieving optimal rhinoplasty outcomes.

## Statement of human and animal rights

As this study is a systematic review based exclusively on previously published research, no new human participants or animals were involved. All data analyzed were derived from publicly available studies.

## Informed consent

No informed consent is required.

## Declaration of competing interest

The authors declare no commercial, financial, institutional, or personal relationships that could be perceived as influencing the design, execution, or interpretation of this research. No conflicts of interest exist that might compromise the integrity or impartiality of the study findings.

## Appendix Supplementary materials

Appendices Supplementary Table 1 - Characteristics of included studies. Image, application 1

Supplementary Table 2 - Outcomes and complications by graft type. Image, application 2

Supplementary Table 3 - Full risk of bias assessment of included studies using ROBINS‑I for non‑randomized studies and RoB 2 for randomized controlled trials. Image, application 3

Supplementary Table 4 - Subgroup trends in outcomes, complications, and regional preferences for autologous and alloplastic grafts in augmentation rhinoplasty. Image, application 4

Supplementary Table 5 - Full-text articles excluded after eligibility review with corresponding reasons for exclusion Image, application 5
