# Supplementary material for: Outcomes and complications of autologous versus alloplastic grafts in augmentation rhinoplasty: A systematic review of studies from 2000 to 2024
Source: JPRAS Open. 2026 Jan 28;51:330–44. doi: 10.1016/j.jpra.2026.01.031 (PMC13396615; doi:10.1016/j.jpra.2026.01.031)
Supplement: Supplementary file 1 — Appendices Supplementary Table 1 - Characteristics of included studies. [file mmc1.pdf]

Supplementary Table 1. Characteristics of Included Studies

First Author	Year	Country	Design	Sample Size	Graft Type	Follow -Up
Aldosari	2023	Saudi Arabia	Case series (Retrospective)	30	Alloplast: No; Autologous: Yes	5
Bhat	2024	India	Retrospective Observational Study	210	Alloplastic: No; Autologous: Yes	1-10
Bullocks	2011	USA	Retrospective case series	68	Alloplastic: No; Autologous: Costal cartilage (7th rib), mixed with minced adipose tissue	0.5-3
Choi	2020	South Korea	Retrospective case series	110	Alloplastic: None; Autologous (source): Rib cartilage (all patients)	5
Ferrill	2013	USA	Case series	15	Autologous: No; Alloplastic: Yes (Silicone, Gore-Tex, Medpor)	0.5-5.5
Fu	2023	China	Case series	25	Alloplastic: No; Autologous: Costal cartilage (7th rib), mixed with minced adipose tissue;	0.5-1.5
Gu	2024	China	Case Series	65	Alloplastic: No; Autologous: Yes	0.5-2
Joo	2016	Republic of Korea	Retrospective Cohort Study	244	Alloplastic: Yes; Autologous: Yes	NR
Kaiser	2024	Switzerland & Germany	Phase 1 Clinical Trial/Case Series	5	Alloplastic: No; Autologous: Yes	1
Khan	2024	Pakistan	Retrospective cohort study	58	Alloplastic: None<br>Autologous: Dermal fat graft;	1
Kim	2014	South Korea	Case series	581	Alloplastic: Yes; Autologous: No	1.5-13
Korn	2024	Germany	Retrospective	21	Alloplastic: No; Autologous: Yes	3-3.5
Liyanage	2020	UK	Retrospective Case Series	100	Alloplastic: No; Autologous: Yes	1-3
Manafi	2015	Iran	Case series	128	Alloplastic: None<br>Autologous: Septal (75%), Rib (20%), Conchal (5%);	1-2.5
Mehta	2021	India	Observational (case series)	12	Autologous: Olecranon bone graft (forearm)	2-5
Moon	2012	South Korea	Retrospective Case Series	108	Alloplastic: No; Autologous: Yes	0.5-5
Qian	2014	United Kingdom	Case series	5	Autologous (source): Rib cartilage	2-3
Rohrich	2022	USA	Retrospective cohort study	226	Alloplastic: Fresh frozen rib cartilage (cadaveric; non-irradiated)	0.5-8
Sayed	2022	Egypt	Retrospective Case Series	32	Alloplastic: No; Autologous: Yes	1-2
Shawky	2024	Egypt	Cross-sectional study	30	Alloplast: No; Autologous: Yes	0.5-1.5
Truong	2025	USA	Retrospective case series	1019	Alloplastic: Silicone (custom-carved grade 40 silicone block); Autologous: Septal or conchal cartilage used in some for-tip projection	1
Varadharajan	2015	India	Case series	21	Alloplastic: No; Autologous: Yes (source: costal cartilage -rib)	0.5
Vila	2014	USA	Retrospective case series	97	Alloplastic: No; Autologous: Yes	1-5
Wee	2017	South Korea	Retrospective Clnical Study	63	Alloplastic: No; Autologous: Yes	1

Widodo	2021	Indonesia	Case report (2 patients) Excluded based on protocol (<10 patients)	2	Alloplastic: No; Autologous: Yes	.5
Winkler	2012	USA	Retrospective cohort study	659	Alloplastic: Yes; Autologous: No	1
Yan	223	China	Randomized Controlled Trial	70	Alloplastic: No; Autologous: Yes	1
Yang	2017	South Korea	Retrospective Case Series	18	Alloplastic: Yes; Autologous: No	0.5-3

Summary of key characteristics of the 28 included studies, including study design, sample size, graft type, and follow-up period.
