# Supplementary material for: Outcomes and complications of autologous versus alloplastic grafts in augmentation rhinoplasty: A systematic review of studies from 2000 to 2024
Source: JPRAS Open. 2026 Jan 28;51:330–44. doi: 10.1016/j.jpra.2026.01.031 (PMC13396615; doi:10.1016/j.jpra.2026.01.031)
Supplement: Supplementary file 2 — Supplementary Table 2 - Outcomes and complications by graft type. [file mmc2.pdf]

Supplementary Table 2. Outcomes and Complications by Graft Type[1]

Study	Total Number of Cases	Infection	Extrusion	Warping	Resorp tion	Revision Rate	Patient Satisfaction
Aldosari	30	0	0	0	2 cases	NR	Good
Bhat	210	1	NR	0	2	2	High
Bullocks	68	1	0	0	0	1	High
Choi	110	0	0	0	0	1	High
Ferril	15	5	4	NR	NR	19	Good
Fu	25	1	0	0	NR	3	High
Gu	65	0	0	NR	NR	0	High
Joo	244	0	0	0	NR	1	High
Kaiser	5	2	0	0	NR	3	High
Khan	58	0	0	NR	NR	0	High
Kim	581	84	21	NR	NR	581	NR
Korn	21	0	0	0	2	1	High
Liyanage	100	0	1	NR	NR	3	High
Manafi	128	0	0	NR	Partial	5	High
Mehta	12	0	0	0	0	1	NR
Moon	108	0	1	0	0	3	High
Qian	5	0	0	0	NR	NR	Good
Rohrich	226	6	0	6	NR	5	NR
Sayed	32	0	0	2 cases	NR	1 case	High
Shawky	30	0	0	NR	0	2	NR
Truong	1019	3	9	NR	NR	103	NR
Varadharajan	21	0	0	2	2	2	Good
Vila	97	0	0	2	0	6	High
Wee	63	0	0	0	0	0	High
Widodo	2	0	0	0	0	1	High
Winkler	659	57	18	NR	NR	NR	NR
Yan	70	0	0	NR	NR	NR	High
Yang	18	0	0	NR	2	2	Good

Outcomes and complications reported in the included studies, categorized by graft type.

[1] *NR = Not Reported. Complication rates are presented as absolute numbers; percentages were calculated relative to the total number of cases per study. Patient satisfaction was categorized as reported in the original articles (e.g., "Good," "High").*
