# Supplementary material for: Outcomes and complications of autologous versus alloplastic grafts in augmentation rhinoplasty: A systematic review of studies from 2000 to 2024
Source: JPRAS Open. 2026 Jan 28;51:330–44. doi: 10.1016/j.jpra.2026.01.031 (PMC13396615; doi:10.1016/j.jpra.2026.01.031)
Supplement: Supplementary file 3 — Supplementary Table 3 - Full risk of bias assessment of included studies using ROBINS‑I for non‑randomized studies and RoB 2 for randomized controlled trials. [file mmc3.pdf]

Supplementary Table 3. Full-Text Articles Excluded after Eligibility Review, with Reasons[1]

First Author	Year	Title	Reason for Exclusion
Aldosari	Case series	ROBINS-I	Moderate
Bhat	Retrospective observational	ROBINS-I	Moderate
Bullocks	Case series	ROBINS-I	Serious
Choi	Case series	ROBINS-I	Moderate
Ferrill	Case series	ROBINS-I	Serious
Fu	Case series	ROBINS-I	Moderate
Gu	Case series	ROBINS-I	Moderate
Joo	Retrospective cohort	ROBINS-I	Moderate
Kaiser	Phase 1 clinical trial	ROBINS-I	Serious
Khan	Retrospective cohort	ROBINS-I	Moderate
Kim	Case series	ROBINS-I	Serious
Korn	Retrospective	ROBINS-I	Moderate
Liyanage	Retrospective case series	ROBINS-I	Moderate
Manafi	Case series	ROBINS-I	Moderate
Mehta	Observational (case series)	ROBINS-I	Moderate
Moon	Case series	ROBINS-I	Moderate
Qian	Case series	ROBINS-I	Moderate
Rohrich	Retrospective cohort	ROBINS-I	Moderate
Sayed	Case series	ROBINS-I	Moderate
Shawky	Cross-sectional study	ROBINS-I	Moderate
Truong	Retrospective case series	ROBINS-I	Moderate
Varadharajan	Case series	ROBINS-I	Serious
Vila	Retrospective case series	ROBINS-I	Moderate
Wee	Retrospective clinical study	ROBINS-I	Moderate
Widodo	Case report (2 patients)	ROBINS-I	Serious
Winkler	Retrospective cohort study	ROBINS-I	Serious
Yan	Randomized controlled trial	RoB 2	Low
Yang	Case series	ROBINS-I	Moderate

Risk of bias assessment for the 28 included studies using ROBINS-I for non-randomized studies and RoB 2 for randomized controlled trials.

[1] *ROBINS-I = Risk Of Bias In Non-randomized Studies – of Interventions; RoB 2 = Cochrane Risk of Bias tool for Randomized Trials. Judgments are presented at the study level.*
