# Supplementary material for: Outcomes and complications of autologous versus alloplastic grafts in augmentation rhinoplasty: A systematic review of studies from 2000 to 2024
Source: JPRAS Open. 2026 Jan 28;51:330–44. doi: 10.1016/j.jpra.2026.01.031 (PMC13396615; doi:10.1016/j.jpra.2026.01.031)
Supplement: Supplementary file 4 — Supplementary Table 4 - Subgroup trends in outcomes, complications, and regional preferences for autologous and alloplastic grafts in augmentation rhinoplasty. [file mmc4.pdf]

Supplementary Table 4. Summary of Subgroup Trends in Augmentation Rhinoplasty Graft Use

Graft Type	Common Complications	Overall Satisfaction Trend	Preferred Regions	Notes
Autologous	Warping	Higher long-term satisfaction, more natural contour	Middle East, Western countries	Often used in revision or complex cases; rib cartilage favored for support
Alloplastic	Infection, Extrusion, Late displacement	Higher early satisfaction; long-term revision more likely	East Asia	Easier to shape and insert; preferred in primary cases requiring less augmentation

Observed trends in complication profiles, satisfaction, and regional preferences between autologous and alloplastic grafts.
