# Supplementary material for: Outcomes and complications of autologous versus alloplastic grafts in augmentation rhinoplasty: A systematic review of studies from 2000 to 2024
Source: JPRAS Open. 2026 Jan 28;51:330–44. doi: 10.1016/j.jpra.2026.01.031 (PMC13396615; doi:10.1016/j.jpra.2026.01.031)
Supplement: Supplementary file 5 — Supplementary Table 5 - Full-text articles excluded after eligibility review with corresponding reasons for exclusion [file mmc5.pdf]

Supplementary Table 5. Full-Text Articles Excluded after Eligibility Review, with Reasons[1]

First Author	Year	Title	Reason for Exclusion[2]
Alhussain	2020	Efficacy of Autologous vs. Homologous Costal Cartilage Grafts in Dorsal Augmentation Rhinoplasty: A Systematic Review and Meta-analysis	Review article
AlQurashi	2023	Costal Cartilage Grafts in Dorsal Augmentation Rhinoplasty: A Systematic Review and Meta-Analysis	Review article
Alamri	2024	Outcomes and complications of closed vs open rhinoplasty: a systematic review	Focus mismatch
Baqudair	2021	Harvesting Costal Cartilage for Secondary Rhinoplasty: Techniques, Considerations, and Outcomes	Review article
Bateman	2000	Retrospective review of augmentation rhinoplasties using autologous cartilage grafts	Old publication (2019)
Dermody	2023	Overview of Implant Options in Rhinoplasty: A Review	Narrative review (not a clinical study)
Fitriana	2024	The Efficacy and Complication Rates of Augmentation Rhinoplasty Using Autologous Cartilage Versus Silicone Implants: A Comprehensive Systematic Review	Duplicate / Secondary review
Genther	2016	Surgical Nasal Implants: Indications and Risks	Narrative review article
Han	2023	Comparison of nasal valve dysfunction treatment outcomes for temperature-controlled radiofrequency and functional rhinoplasty surgery: a systematic review and meta-analyses	Focuses on nasal valve dysfunction treatments
Harutyunyan	2024	Dorsal Augmentation Rhinoplasty by Cartilage Allograft	Wrong graft type focus
Harutyunyun	2024	Use Of Grafts Materials in Dorsal Augmentation Rhinoplasty	Review article summarizing graft options
Ho	2019	Rhinoplasty outcomes and trends	Review article
Hosseini	2025	A comprehensive overview of FFRG and IHCC allograft cartilages in revision rhinoplasty: a systematic review	Review article
Hudise	2022	Complications of the nasal dorsum reconstruction using autologous or alloplastic grafts: evidence from systematic review and meta-analysis	Review article

[1] *Reasons for exclusion were based on prespecified criteria, including focus mismatch, review or guideline status, wrong intervention, duplicate publication, or insufficient original clinical data.*

Hwnag	2024	Complicated calcified alloplastic implants in the nasal dorsum: A clinical analysis	Wrong focus
Ishii	2017	Clinical Practice Guideline: Improving Nasal Form and Function after Rhinoplasty	Guideline paper
Kao	2015	Microautologous Fat Transplantation for Primary Augmentation Rhinoplasty: Long-Term Monitoring of 198 Asian Patients	Focuses on microautologous fat transplantation
Keyhan	2022	Prevalence of complications associated with polymer-based alloplastic materials in nasal dorsal augmentation: a systematic review and meta-analysis	Review article
Keyhan	2021	A Systematic Review and Meta-Analysis of Complications Associated with Autogenous Diced Cartilage Wrapped in Fascia Used in Nasal Dorsum Augmentation	Review article
Kim	2015	Rhinoplasty with Cartilage and Alloplastic Materials, Nasal SMAS Management in Asian Rhinoplasty, Contracture Classification, and Secondary Rhinoplasty with Contracture	Narrative review / expert opinion
Li	2021	Long-Term Complications from Diced Cartilage in Rhinoplasty: A Meta-analysis	Wrong intervention focus
Loyo	2013	Safety of Alloplastic Materials in Rhinoplasty	Review article
Na	2017	Dorsal Augmentation using Alloplastic Implants	Descriptive surgical technique article
Nguyen	2015	Simple Implant Augmentation Rhinoplasty	Narrative review
Nikparto	2024	The current techniques in dorsal augmentation rhinoplasty: a comprehensive review	Focus mismatch
Nocini	2023	Proposal for Perioperative Pharmacological Protocol for the Reduction in Early Complications in Orthorhinoseptoplasty: Five Years of Experience	Focuses on pharmacological perioperative protocols
Orhan	2013	The use and possible complications of graft materials in rhinoplasty	Invited review article
Rettinger	2007	Risks and complications in rhinoplasty	Review article
Shafik	2025	Outcomes and complication rates of different materials used for nasal dorsal augmentation in septorhinoplasty: a systematic review and meta-analysis	Secondary systematic review/meta-analysis
Won	2019	Narrative Review of Autologous Techniques in Asian Rhinoplasty	Narrative review (no original data)
Nandakumar	2022	Grafts in Septorhinoplasty: a systematic review and future directions	Review article
Wu	2022	A systematic review and meta-analysis of the efficacy and complication rates of augmentation	Review article

		rhinoplasty with autologous cartilage and silicone prosthesis	

Full-text articles excluded after eligibility review, with reasons for exclusion according to predefined criteria.
